# Progress in Obstetrics

**Published:** 1898-05-28

**Authors:** 


### PROGRESS IN OBSTETRICS.

(*Continued from page* 48.)

**Danger of Pregnancy after Ovariotomy.**—That a certain amount of risk occurs in pregnancy and labour after ovariotomy is well known, although a large number of cases occur without accident. One example may be given of a possible danger in labour, namely, the possibility of rupture of an adhesion from uterine contraction with fatal intra-peritoneal hæmorrhage. The following case, reported by Laroyenne,[8] illustrates a risk in pregnancy. An easy ovariotomy was performed on a young multipara, from which she recovered without any bad symptom. One menstrual period occurred after the operation, and she then became pregnant. During the second month of pregnancy she was seized with severe abdominal pains, accompanied by dyspnœa and vomiting. Five days later she was admitted to hospital in a moribund condition, and died the following day. At the autopsy the peritoneal cavity was found to be filled with blood clot. It was found that, owing to traction, caused by the growth of the uterus, one of the ligatures had slipped from the stump, thus allowing fatal hæmorrhage to occur.

**Results of Ovariotomy for Malignant Tumours.**—Kratzenstein[9] reports the results of operations in 100 cases of malignant disease of the ovary in the Berlin Clinic. The series included cases of both carcinoma and sarcoma, and the growths were both primary and secondary. The series commenced in 1879, and was completed in 1892. Twenty-eight cases died, 11 of these deaths being due to sepsis. Of the remainder the disease recurred in 34, and all but two of these had terminated fatally. No recurrence occurred in any of the cases of fibro-sarcoma. Of the total number of cases, 36 per cent. were well at periods varying from five and a half to fourteen and a half years after operation. These results justify the removal of malignant tumours of the ovary, but, in performing the operation, if the best results are to be obtained, great care should be exercised to avoid the inoculation of healthy tissue with particles of the malignant growth by making a sufficiently large incision previous to puncturing the cyst, if this be necessary, and by using the greatest care and gentleness in separating adhesions so as to avoid tearing the tumour. The importance of early diagnosis and operation cannot be too much emphasized. The absence of recurrence in the cases of fibro-sarcoma is considered by the author to show that these growths are not malignant. It is unfortunate that the series was commenced at a date when the lessening of mortality from sepsis which has occurred in later years had not reached its present degree; it is very probable that, of the 11 deaths from sepsis, the majority took place before 1886, and the mortality from this cause would probably now be much less. The mortality from other causes is not likely to be much diminished, except by earlier diagnosis and operation.

**Post-Typhoid Suppuration in Ovarian Cyst.**—The danger of the occurrence of suppuration in an ovarian cyst existing in a patient suffering from typhoid must always be remembered; hectic and other symptoms of suppuration, arising after the subsidence of the fever and apparent recovery, should lead to an examination of the condition of the contents of tumour after abdominal section.

Petha[10] reports a case of a patient suffering from an ovarian cyst who developed typhoid fever. Four months after the accession of the fever the cyst was removed and found to be suppurating. Examination of the contents showed that the typhoid bacilli were present in the cyst in large numbers. It appeared that the bacilli had migrated through the cyst wall and produced the suppuration as a metastatic process. The existence of the bacilli after such a long interval shows their vitality, and also indicates that the contents of such a cyst are a favourable culture medium. Other cases are reported in which pure cultures of the bacillus were found eight months after recovery. It is not certain that the bacilli were the direct cause of the suppuration, although there is little doubt of their pyogenic action. The occurrence of pelvic abscess and other suppurations after typhoid is of course well known.

[8] Amer. Jour. Med. Sci., January, 1898. [9] Ditto. [10] Ditto.

